# Prospective comparative study of scaphocapitate arthrodesis with and without lunate excision for stage IIIB Kienböck’s disease

**DOI:** 10.1007/s00402-025-06147-6

**Published:** 2025-12-18

**Authors:** Galal Hegazy, Mahmoud Seddik, Rashed El-Sadek, Mohamed Gamal, Elsayed Shaheen, Mohammed Alnahas, Ibrahem El-Sebaey, Abdulhamid Elzoghby, Ahmed Darweash

**Affiliations:** 1 Al Azhar University, Cairo, Egypt; 2 Suez University, Suez, Egypt

**Keywords:** Kienböck’s disease, Scaphocapitate arthrodesis, Lunate preservation, Wrist fusion, Avascular necrosis, Carpal alignment

## Abstract

**Introduction:**

Kienböck’s disease is a progressive avascular necrosis of the lunate that causes wrist pain and functional impairment. Surgical intervention is often needed for advanced stage IIIB disease to relieve symptoms and preserve the carpal structure. Scaphocapitate (SC) arthrodesis is an established technique, but the value of lunate excision versus preservation remains debated. This study prospectively compared the outcomes of SC arthrodesis performed with and without lunate excision.

**Materials and methods:**

This prospective comparative study included 38 patients with stage IIIB Kienböck’s disease, who were allocated to receive SC arthrodesis with lunate preservation (*n* = 19) or lunate excision (*n* = 19). The follow-up time was 84 months. The primary outcome measure was pain intensity, which was assessed via the visual analogue scale (VAS) for pain. The secondary outcomes included wrist function evaluated by the modified Mayo wrist score (MMWS) and patient-rated wrist evaluation (PRWE), grip strength, wrist range of motion (ROM), and radiographic parameters such as ulnar variance, the carpal height ratio (CHR), the carpal ulnar distance (CUD), and the radioscaphoid (RS) angle. The union rate, time to union, time to return to work, and incidence of postoperative complications were also recorded.

**Results:**

Both groups demonstrated significant postoperative improvement in pain (VAS pain score, *p* = 0.001). At 84 months, the preservation group achieved superior pain control, with a lower mean VAS score (5 versus 12, *p* = 0.001). The preservation group also maintained a greater CUD (30 mm versus 23 mm, *p* = 0.001) and exhibited later onset of degenerative changes. MMWS improved from 49 to 73 in the preservation group and from 48 to 72 in the excision group. The PRWE scores decreased from 69 to 21 in the preservation group and from 67 to 24 in the excision group. The final grip strength reached 88% of that of the contralateral side in the preservation group and 84% in the excision group, with no significant difference in ROM. The union rates were 93% for preservation and 91% for excision. The excision group presented higher rates of degenerative changes (16 versus 8 cases) and nonunion (2 versus 1). No major differences were found in the infection rate, sympathetic reflex dystrophy, or return to work.

**Conclusions:**

SC arthrodesis provides reliable long-term pain relief in patients with stage IIIB Kienböck’s disease. Lunate preservation provides superior pain control and carpal alignment, reduces degenerative sequelae, and is recommended where feasible.

## Introduction

Kienböck’s disease, also known as avascular necrosis of the lunate, is an uncommon and progressive condition resulting from disruption of the lunate’s vascular supply. This vascular insufficiency leads to osteonecrosis, subchondral collapse, and the eventual development of degenerative changes within the wrist joint [1]. The condition most frequently affects individuals between the second and fourth decades of life, with a male predominance, and typically presents with chronic wrist pain, a reduced range of motion (ROM), and functional impairment [2].

The aetiology of Kienböck’s disease is multifactorial and not yet fully understood, with contributions from anatomical, biomechanical, and biological factors. Ulnar negative variance has been implicated in increasing axial load transmission through the lunate, whereas the tenuous vascular anatomy of the bone is particularly susceptible to ischaemic insult. Additional hypotheses suggest that venous congestion, intraosseous compartment syndrome, and localised inflammatory processes may contribute to disease onset and progression [3].

**Fig. 1:**
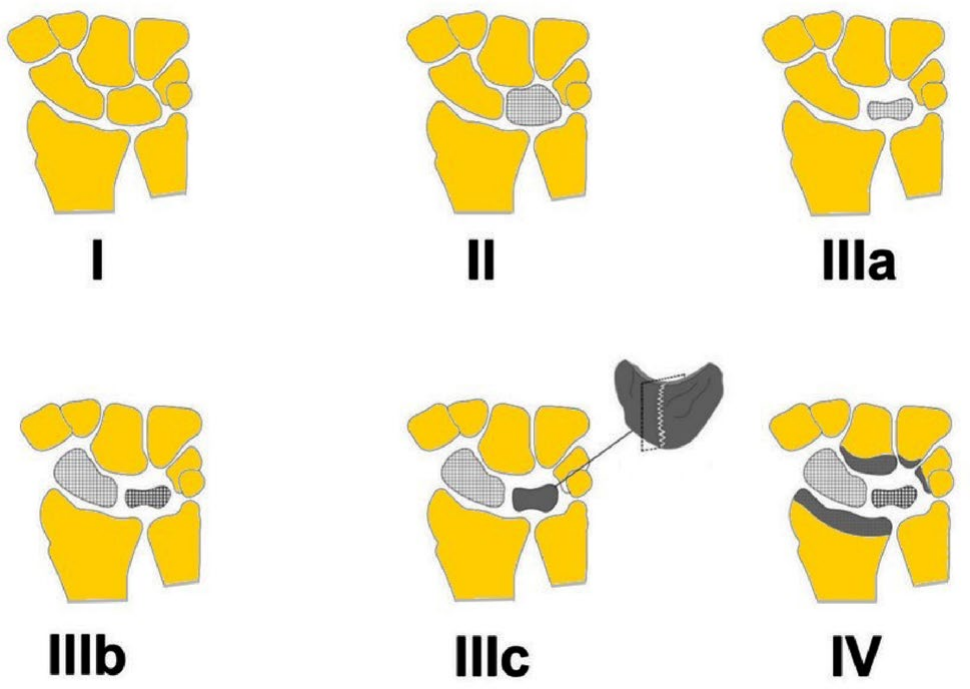
Lichtman classification stages of Kienböck’s disease. Illustration demonstrating the progressive stages (I–IV) of lunate avascular necrosis. Stage IIIB (bottom left) involves lunate collapse with fixed carpal malalignment, the main focus of the present study

Disease severity is most commonly assessed via the Lichtman classification system, which stratifies radiographic findings into discrete stages to guide treatment selection [4, 5] (Fig. 1). In advanced stages, particularly stages IIIB and IIIC, surgical management is typically required to alleviate pain, offload the compromised lunate, and preserve carpal integrity [6, 7].

Scaphocapitate (SC) arthrodesis is a recognised surgical technique in this context that involves the fusion of the scaphoid and capitate bones to reduce mechanical loading through the lunate. This procedure provides midcarpal stabilisation while preserving partial wrist motion. However, the necessity and clinical benefit of concomitant lunate excision during SC arthrodesis remain controversial [8]. Proponents of lunate excision argue that removal of the necrotic bone eliminates a potential pain generator and prevents further collapse [9–12]. Conversely, others advocate for lunate preservation, suggesting that it helps maintain carpal alignment, avoids proximal row instability, and reduces the risk of progressive degenerative changes in adjacent joints [13–16].

Despite the range of surgical options available for advanced Kienböck’s disease, there remains a paucity of high-level comparative evidence assessing the functional and radiological outcomes of SC arthrodesis with or without lunate excision. The present study was designed to address this gap by directly comparing these two surgical strategies in patients with stage IIIB Kienböck’s disease. The primary objective was to compare pain levels between the treatment groups via the visual analogue scale (VAS). The secondary objectives included evaluating functional outcomes via the modified Mayo wrist score (MMWS) and patient-rated wrist evaluation (PRWE) and documenting the incidence of postoperative complications such as nonunion, hardware failure, adjacent joint arthritis, and postoperative carpal malalignment. We hypothesised that SC arthrodesis with lunate preservation would yield superior clinical and radiological outcomes compared with SC arthrodesis combined with lunate excision.

## Materials and methods

This study was approved by the Institutional Review Board of our university hospitals. Written informed consent was obtained from all participants in accordance with the Declaration of Helsinki.

Eligible participants were adults aged 18 years or older with a confirmed diagnosis of stage IIIB Kienböck’s disease. The diagnosis was based on standard posteroanterior and lateral wrist radiographs, supported by magnetic resonance imaging (MRI) and computed tomography (CT) scans. The exclusion criteria included bilateral wrist involvement, follow-up shorter than 36 months, prior wrist surgery, or a history of inflammatory arthritis.

**Fig. 2:**
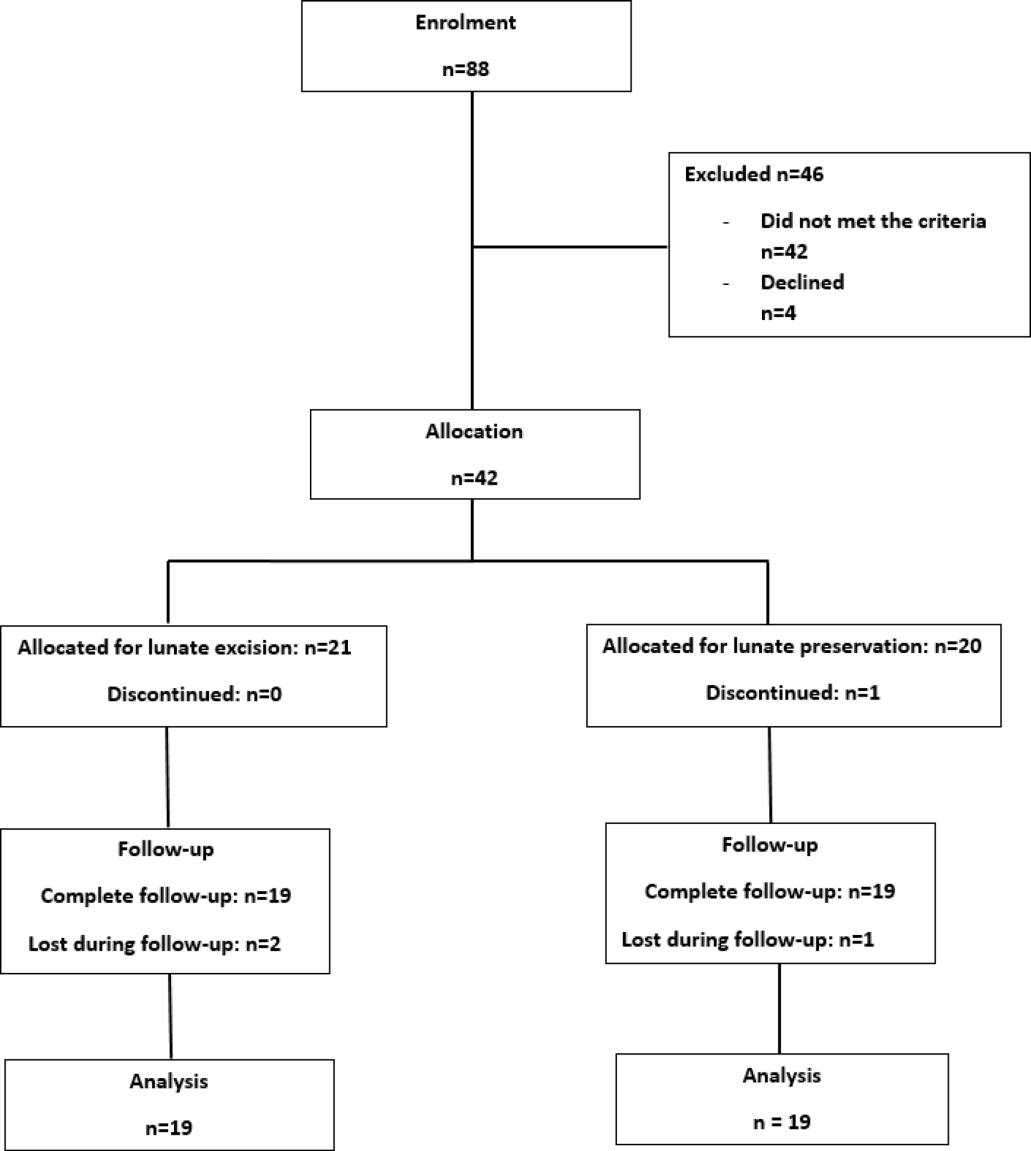
CONSORT flow diagram of patient recruitment and allocation

A total of 88 patients with Kienböck’s disease were initially screened for eligibility. Forty-six patients were excluded—42 did not meet the inclusion criteria, and four declined to participate between February 2015 and December 2024. The remaining 42 eligible patients were randomised into two groups via a computer-generated allocation schedule. Twenty-one patients were assigned to undergo SC arthrodesis with lunate excision, and twenty patients were assigned to undergo SC arthrodesis with lunate preservation. In the preservation group, one patient discontinued participation, whereas in the excision group, all patients completed the intervention. During follow-up, one patient from the preservation group and two patients from the excision group were lost to follow-up. Consequently, 38 patients (19 in each group) completed the study and were included in the final analysis, as detailed in the CONSORT flow diagram (Fig. 2).

### Outcome measures

Clinical and radiographic assessments were performed preoperatively and at 18, 36, 60, and 84 months post-operatively. The pain intensity was evaluated via the VAS score for pain [17], ranging from 0 to 100 mm. The degree of pain severity was categorised as follows: 0 (no pain), ≤ 19 mm (very mild), 20–35 mm (mild interference), 36–60 mm (moderate interference), and ≥ 60 mm (severe interference).

Total wrist ROM, including flexion–extension, radial–ulnar deviation, and pronation–supination, was measured with a two-hand goniometer, and the results are expressed as a percentage of the contralateral, healthy wrist [18]. Maximal grip strength was measured via a Jamar hand dynamometer [19], expressed as a percentage of the contralateral side and adjusted for hand dominance. Functional status was assessed via the MMWS [20] and the PRWE score [21].

**Fig. 3:**
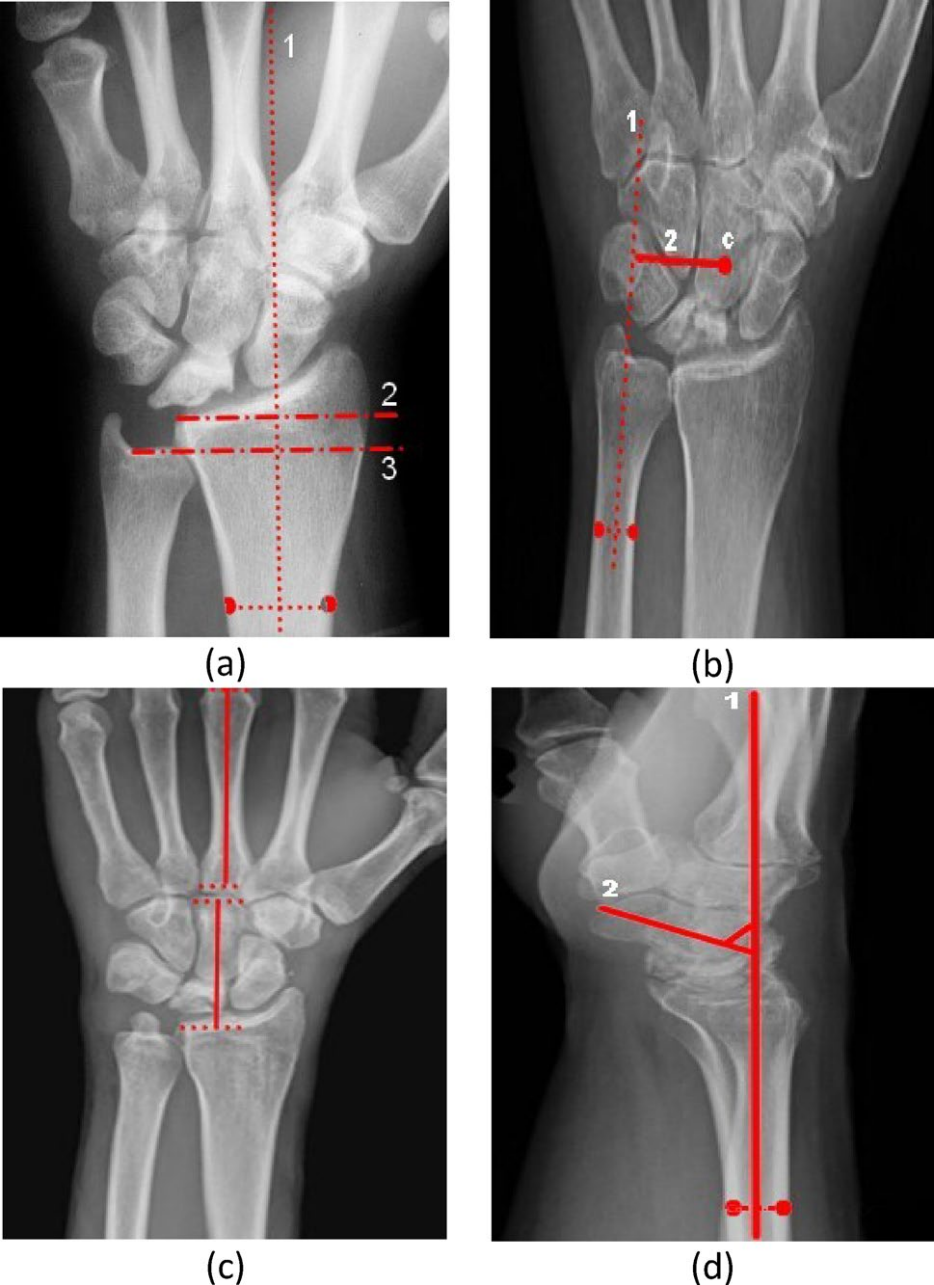
Ulnar variance was calculated as the perpendicular distance between the distal ulnar edge and a reference line drawn from the distal articular surface of the radius The CUD is defined as the distance between the ulnar border of the lunate and the perpendicular line from the radial styloid and is used to assess carpal translocation CHR was calculated via Youm’s method as the ratio of carpal height to the length of the third metacarpal axis—an indicator of carpal collapse The RSA is calculated as the angle between the longitudinal axis of the radius and a line along the scaphoid

Radiographic parameters [22] included ulnar variance (measured via perpendicular lines, Fig. 3a), carpal ulnar distance (CUD, Fig. 3b), the carpal height ratio (CHR, Fig. 3c) calculated via Youm’s method, and the radioscaphoid (RS) angle (Fig. 3d). The degree of osteoarthritis in the radioscaphoid and scaphotrapeziotrapezoid joints was graded according to the Kellgren–Lawrence criteria [23].

To maintain unbiased and objective outcome assessment, independent orthopaedic teams were assigned to evaluate each treatment group. Two orthopaedic surgeons independently assessed the clinical outcomes in the excision group, while a separate pair evaluated the preservation group. Radiographic evaluations were similarly performed by two independent surgeons for each group. All assessors were blinded to treatment allocation and to the study aims and hypotheses. Although patients and surgical teams were aware of the procedure performed, potential bias was mitigated by ensuring identical management protocols, follow-up schedules, and blinded outcome assessments across both groups.

### Surgical technique

All procedures were performed under regional anaesthesia with the application of an upper arm pneumatic tourniquet. Patients were placed in the supine position, and surgeries were conducted by a senior surgeon or under direct supervision. A mid-dorsal skin incision was made, extending from the base of the third metacarpal to 1 cm proximal to the dorsal ridge of the distal radius. The extensor retinaculum was incised between the third and fourth extensor compartments, and the posterior interosseous nerve was identified and denervated. A longitudinal incision was made in the dorsal joint capsule to allow inspection of the carpal bones and their articular surfaces. In the excision group, the lunate was excised, whereas in the preservation group, the lunate was preserved. The articular surfaces of the scaphoid and capitate were then prepared by removing the articular cartilage, except for the volar rim, to maintain the space between the scaphoid and capitate bones. Kirschner wires were utilised as joysticks to correct carpal misalignment, facilitating the release and derotation of the scaphoid to restore the RS angle between 40° and 60°. Temporary stabilisation of the SC joint was achieved via Kirschner wires. A cancellous bone graft, harvested from the distal radius, was inserted into the prepared site. The SC joint was fixed under fluoroscopic guidance via two 3-mm Herbert compression screws, ensuring that excessive compression at the arthrodesis site was avoided. Following tourniquet deflation and haemostasis, the wound was thoroughly irrigated and closed in layers. A sterile dressing was applied, and the wrist was immobilised in a short-arm thumb spica cast, positioned in slight extension and neutral deviation.

### Postoperative management

**Fig. 4:**
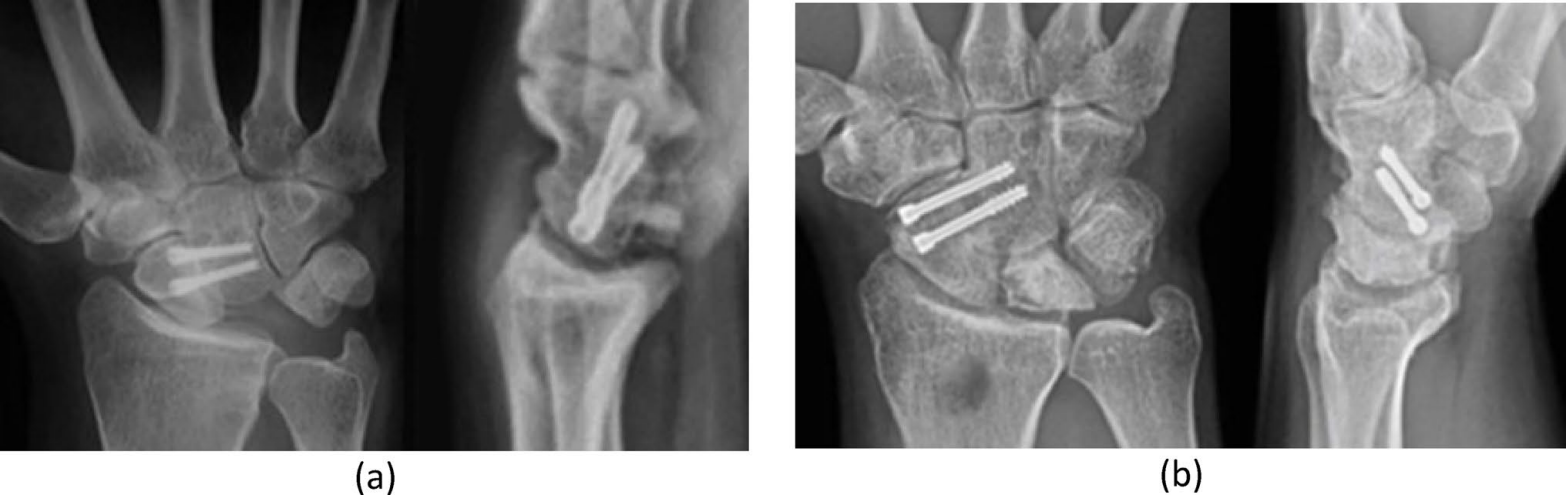
Postoperative radiographs after SC arthrodesis with lunate excision. Anteroposterior and lateral views showing scaphocapitate fusion and removal of the necrotic lunate Postoperative radiographs after SC arthrodesis with lunate preservation. Anteroposterior and lateral views showing preserved lunate

Postoperative care included pain management and elevation of the hand to minimise swelling. Sutures were removed two weeks after surgery, and a waterproof fiberglass thumb spica cast was applied for six weeks. Patients were then provided with a removable splint until radiographic confirmation of union at the arthrodesis site. A structured rehabilitation program, supervised by a specialised hand physiotherapist, was initiated immediately after surgery to restore wrist, elbow, and shoulder function. Radiographic follow-ups, including standard posteroanterior and lateral wrist views, were performed every two weeks to monitor union. Union was defined as the absence of gaps or lucency at the arthrodesis site, with visible trabecular bone bridging (Fig. 4). If union was uncertain, a CT scan was conducted every three weeks. Nonunion was diagnosed if less than 50% trabecular bone bridging was observed on CT scans at 24 weeks post-surgery. Patients with sedentary jobs were permitted to return to work gradually while still in the cast, whereas manual laborers were advised to resume work only after union was confirmed, with activities limited by pain tolerance. Full return to work and unrestricted activities were allowed once union was achieved and pain was adequately controlled.

### Statistical analysis

A sample size calculation was performed to ensure adequate statistical power to detect differences in the primary outcome measure (VAS pain score). A minimum of 17 patients per group were required to achieve 90% power at a significance level of *p* < 0.05. Continuous variables were assessed for normality via the Shapiro–Wilk test. Between-group comparisons were performed via the independent-samples *t*-test or Mann–Whitney *U* test, as appropriate. Categorical variables were compared via the chi-square test or Fisher’s exact test. Repeated measures within each group were analysed via repeated-measures ANOVA for normally distributed data or the Friedman test for nonparametric data. Effect sizes (Cohen’s *d*) with 95% confidence intervals (CIs) were calculated for key between-group comparisons to quantify the magnitude of differences. The results are presented as the means ± standard deviations (SDs) with 95% CIs, and a *p*-value < 0.05 was considered statistically significant. Statistical analyses were conducted on a per-protocol basis, including only patients who completed the full follow-up period; those who withdrew or were lost to follow-up were excluded from the final analysis.

## Results

At baseline, there were no significant demographic or clinical differences between the groups (Table 1).

**Table 1:** **Demographics data for excision vs. preservation** data are reported as mean ± SD with a 95% CI

Item	stage IIIB (*n* = 38)		
	Excision (*n* = 19)	Preservation (*n* = 19)	*p* value
Age in years (Mean ± SD)	30 ± 8	32 ± 7	0.388
Gender • Male/Female	14/5	16/3	0.648
Occupation • Manual worker/office workers/student/housewife	9/5/2/3	11/5/1/2	0.427
Affected side • Right/Left	13/6	15/4	0.669
Dominance • Dominant/nondominant	13/6	15/4	0.669
Smoking • Yes/no	4/15	3/16	0.638

### Clinical outcomes

**Fig. 5:**
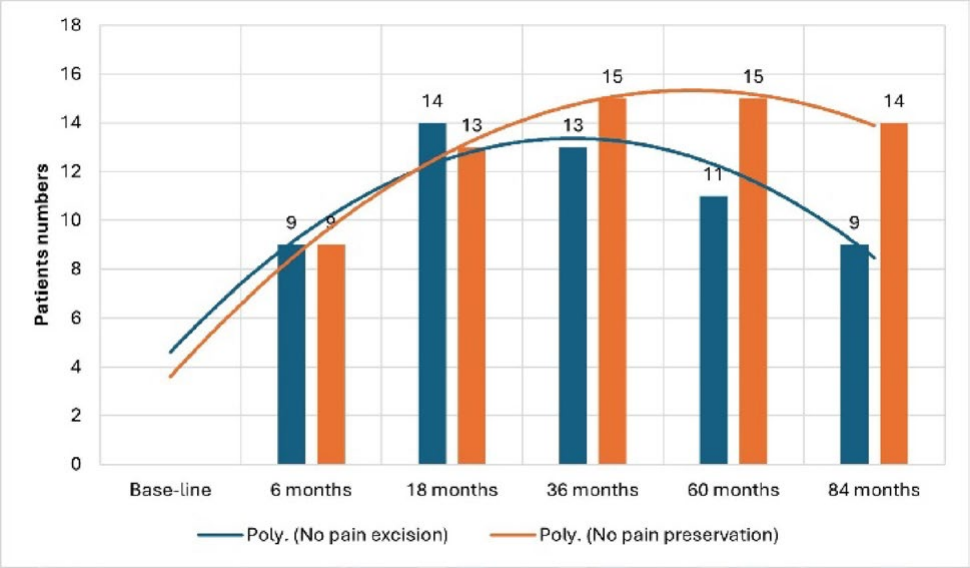
Number of pain-free patients over time. Line chart showing the number of patients reporting no pain (VAS = 0) at each time point up to 84 months. The pain-free rates were consistently greater in the preservation group

Both groups demonstrated significant improvements in pain, motion, strength, and function throughout the follow-up period. The VAS score decreased markedly in both groups, with greater late pain reduction in the preservation group (*p* = 0.001; Cohen’s *d* = 0.68, 95% CI – 0.01 to 1.38) (Table 2). From 36 months onwards, patient numbers without pain remained consistently higher in the preservation group compared to the excision group (Fig. 5). ROM and grip strength improved significantly within groups, reaching comparable values between groups. The functional scores (MMWS and PRWE) improved substantially from baseline, with negligible between-group differences at the final follow-up (MMWS *d* = − 0.13, 95% CI − 0.80 to 0.54; PRWE *d* = 0.34, 95% CI − 0.34 to 1.02) (Table 2).

**Table 2:** Pre- and postoperative clinical outcome measures of excision vs. preservation groups

Outcomes	Baseline	6 months	18 months	36 months	60 months	84 months	*p* value*
VAS pain score (mm)							
Excision	76 ± 7.8	18 ± 14.6	3 ± 2.9	4 ± 5.8	11 ± 8.8	12 ± 8.8	0.001
Preservation	83 ± 8.8	13 ± 14.6	5 ± 4.9	3 ± 1.9	3 ± 2.9	5 ± 2.9	0.001
p value**	0.707	0.317	0.248	0.177	0.001	0.001	
ROM (% of contralateral side)							
Excision	52 ± 1.2	53 ± 1.5	53 ± 1.2	53 ± 1.9	52 ± 1.9	52 ± 1.9	0.421
Preservation	52 ± 1.5	54 ± 1.7	54 ± 1.7	54 ± 1.4	54 ± 1.4	54 ± 1.0	0.532
p value**	0.510	0.598	0.457	0.351	0.132	0.311	
Grip strength (% of contralateral side)							
Excision	50 ± 2.9	71 ± 7.1	89 ± 2.9	89 ± 3.9	86 ± 6.8	84 ± 3.4	0.001
Preservation	51 ± 2.9	73 ± 6.1	89 ± 3.4	89 ± 2.9	89 ± 3.4	88 ± 2.4	0.001
p value**	0.821	0.368	0.673	0.765	0.423	0.194	
MMWS							
Excision	48 ± 6.8	71 ± 4.9	73 ± 3.9	73 ± 2.5	72 ± 4.0	72 ± 4.9	0.001
Preservation	49 ± 6.8	71 ± 4.9	73 ± 2.9	73 ± 3.2	73 ± 2.9	73 ± 2.4	0.001
p value**	0.792	0.872	0.896	0.812	0.759	0.561	
PRWE							
Excision	67 ± 8.8	51 ± 7.1	21 ± 4.4	21 ± 6.8	23 ± 4.4	24 ± 6.3	0.001
Preservation	69 ± 7.8	52 ± 10.9	20 ± 6.1	20 ± 6.0	20 ± 6.1	21 ± 4.9	0.001
p value**	0.786	0.679	0.784	0.682	0.577	0.534	

Data are reported as mean ± SD with a 95% CI. VAS = visual analogue scale for pain (mm); ROM = range of motion (% of contralateral side); MMWS = Modified Mayo Wrist Score; PRWE = Patient-Rated Wrist Evaluation, *p* value*= repeated-measures ANOVA, *p* value**=independent t-test

### Radiographic outcomes

Radiographic parameters remained largely stable in both groups, except for a significant decrease in CUD following lunate excision (*p* = 0.001; *d* = 0.91, 95% CI 0.53 to 1.28). Ulnar variance, the CHR, and the RS angle showed no significant between-group differences at any interval (Table 3).

**Table 3:** Pre- and postoperative radiographic outcome measures of excision vs. preservation

Outcomes	Baseline	6 months	18 months	36 months	60 months	84 months	*p* value*
Ulnar variance (mm)							
Excision	–0.27 ± 3	–0.26 ± 4	–0.27 ± 3	–0.27 ± 3	–0.26 ± 3	–0.27 ± 3	0.679
Preservation	–0.25 ± 3	–0.26 ± 3	–0.27 ± 3	–0.28 ± 3	–0.26 ± 3	–0.26 ± 3	0.598
p value**	0.854	0.828	0.912	0.784	0.867	0.675	
CUD (mm)							
Excision	31 ± 2	29 ± 2	27 ± 2	25 ± 2	24 ± 2	23 ± 2	0.001
Preservation	31 ± 1.8	31 ± 1.7	31 ± 1.9	30 ± 1.7	30 ± 1.7	30 ± 1.8	0.647
p value**	0.785	0.548	0.453	0.031	0.001	0.001	
CHR							
Excision	0.44 ± 0.02	0.44 ± 0.02	0.44 ± 0.02	0.43 ± 0.02	0.43 ± 0.02	0.42 ± 0.02	0.281
Preservation	0.44 ± 0.01	0.44 ± 0.02	0.44 ± 0.02	0.44 ± 0.01	0.44 ± 0.02	0.44 ± 0.02	0.429
p value**	0.648	0.765	0.654	0.438	0.384	0.341	
RS angle (°)							
Excision	58 ± 3	49 ± 5	50 ± 2	50 ± 2	50 ± 2	52 ± 2	0.001
Preservation	59 ± 3	50 ± 3	51 ± 2	50 ± 2	51 ± 2	50 ± 3	0.001
p value**	0.819	0.761	0.769	0.674	0.519	0.612	

Data are reported as mean ± SD with a 95% CI. CUD = carpal ulnar distance (mm); CHR = carpal height ratio; RS angle = radioscaphoid angle (°). *p* value*= repeated-measures ANOVA, *p* value**=independent t-test

The operative time was significantly longer in the excision group (*p* = 0.001); however, the union time, union rate, and return to work did not differ between the groups. Both groups experienced occasional minor complications, including superficial infection, scar sensitivity, reflex sympathetic dystrophy, nonunion, and impingement. Degenerative changes were observed more frequently following lunate excision (Table 4).

**Table 4:** Follow up and complications of stage IIIB excision vs. preservation

Item	Excision (*n* = 9)	Preservation (*n* = 19)	*p* value
Time of surgery (minutes)	75 ± 5	60 ± 7	0.001
Time to union (weeks)	11.5 ± 3	10.8 ± 3	0.582
Union rate % (n)	91 (14)	93 (16)	0.257
Time to full return to work (weeks)	24 ± 5	23 ± 3	0.591
Complications			
• Infection	1	1	0.648
• Sensitive scare	1	1	
• Reflex sympathetic dystrophy	2	1	
• Nonunion	2	1	
• Scaphoid impingement	1	3	
• Degenerative changes	16	8	

Data are reported as mean ± SD with a 95% CI

## Discussion

SC arthrodesis remains a widely accepted salvage procedure for advanced Kienböck’s disease, particularly stage IIIB disease, owing to its ability to offload the compromised lunate by redirecting axial forces through the radial column [24]. While this technique preserves carpal height and reduces stress on the scapholunate ligament, it typically results in some restriction of wrist mobility. The present study evaluated the long-term outcomes of SC arthrodesis performed with and without lunate excision. Both techniques achieved meaningful and sustained improvements in pain, grip strength, and functional scores (VAS, MMWS, and PRWE). However, lunate preservation consistently resulted in superior pain relief and better maintenance of carpal alignment after 60 months. At 60 months, the mean VAS score was 3 mm in the preservation group compared with 11 mm in the excision group, and at 84 months, it was 5 mm versus 12 mm (*p* = 0.001). These findings align with and extend those of previous reports. Hegazy et al. [14] reported similar benefits of lunate preservation in a prospective series of patients with stage IIIB and IIIC disease, where VAS scores improved from 8.2 to 1.3 at a mean follow-up of 45.6 months, and carpal geometry remained stable (RS angle: 59° to 50°; CHR: 0.44 to 0.46). Our findings corroborate these results over a longer follow-up period, demonstrating stable carpal height and improved RS angles in the preservation group, together with a significantly greater CUD (30 mm vs. 23 mm, *p* = 0.001), indicating reduced ulnar translocation.

In contrast, patients who underwent lunate excision experienced worsening pain, which correlated with earlier degenerative changes. These findings are consistent with those of Charre et al. [9], who evaluated SC arthrodesis with lunate excision and reported that pain recurrence in several patients over time was linked to progressive degenerative joint changes. While the CHR remained stable in their series, the reduction in the CUD (from 31 mm to 23 mm, *p* = 0.021) signalled increased ulnar migration. Degenerative changes affected 23% at the RS joint and 9% at the STT joint. Similarly, our excision group exhibited biomechanical deterioration over time, including increased RS angles and earlier onset of arthritic changes (mean onset: 48 months vs. 72 months in the preservation group). Our findings are also in agreement with those of Koh et al. [10], who demonstrated that SC arthrodesis without lunate excision, especially when performed arthroscopically, preserved carpal alignment and resulted in lower VAS scores at 36 months. These data further support the associations among lunate excision, ulnar translocation, and midcarpal instability. While a recent systematic review by Bouri et al. [8] revealed no significant differences in short- to midterm outcomes (≤ 36 months) between preservation and excision, our results highlight that the benefits of lunate preservation become increasingly evident with extended follow-up. The contrasting results of Elshahhat et al. [25] merit discussion. In their retrospective study of 25 patients with stage III disease, lunate excision was associated with superior pain reduction, grip strength, and ROM compared with lunate preservation. At first glance, these results appear to oppose our findings. However, the shorter follow-up in their cohort (≤ 5 years) may explain this discrepancy, as our data show that degenerative progression and worsening pain become evident in the excision group after 60 months. It is therefore plausible that excision provides early symptomatic benefits, whereas preservation confers more durable long-term protection against biomechanical deterioration and ulnar translocation.

Although the incidence of radiographic adjacent joint arthritis was similar between the groups in our study (13% in the excision group vs. 11% in the preservation group; *p* = 0.81), the timing differed significantly. The earlier onset of arthritis in the excision group (mean: 48 months) likely contributed to worsening pain and reduced long-term outcome scores. Importantly, our overall arthritis rates remain lower than those reported in previous studies, such as Luegmair et al. [24], who reported arthritis in up to 35% of patients following SC arthrodesis with lunate excision, and other series with rates exceeding 50% at long-term follow-up [8]. The union rates in our cohort were favourable and consistent across both groups (93% preservation vs. 91% excision), falling within or below the 12–22% nonunion range reported in meta-analyses [8, 15]. This outcome likely reflects the use of Herbert compression screws and autologous bone grafting. However, our nonunion rate was slightly higher than those reported in fully arthroscopic series, where rates approaching zero have been achieved [10, 26]. Grip strength improved significantly in both groups, reaching 84% of the contralateral side in the excision group and 88% in the preservation group at the final follow-up. These results are consistent with those of Hegazy et al. [14], who reported an 84% recovery in a preservation-only cohort. Charre et al. [9] reported a mean grip strength of 34.3 kg, and Elshahhat et al. [25] reported an average grip strength of 28.9 kg. In their meta-analysis, Bouri et al. [8] reported a pooled mean gain of 13.29 kg, translating to a 53% relative improvement. Romeih et al. [27], using sphygmomanometric assessment, demonstrated that stronger grip correlated with improved clinical outcomes, although quantitative values were not reported. Within this context, our results—particularly the 80% strength recovery in the preservation group—represent one of the highest reported levels of grip strength restoration following SC arthrodesis. As expected, in the context of intercarpal fusion, wrist ROM was reduced compared with that of the contralateral wrist but remained functionally adequate. At 84 months, the flexion–extension arc reached 52% in the excision group and 54% in the preservation group. These findings are comparable to those of Charre et al. [9], who reported arcs of 48–54°, and Hegazy et al., who reported 56% preservation in the preservation group. Elshahhat et al. [25] reported a mean postoperative arc of 50.7°, whereas Bouri et al. [8] reported mean flexion and extension values of 33.5° and 38.1°, respectively. Although Romeih et al. [27] highlighted the benefit of arcs ≥ 70° on QuickDASH scores, such mobility is rarely achieved after intercarpal fusion. Nonetheless, the ROM observed in our study was sufficient for daily activities and did not deteriorate over time, confirming that the mechanical limitations of SC arthrodesis do not preclude functional use. The functional scores (MMWS and PRWE) showed robust improvement over time. The MMWS increased from 48 to 49 preoperatively to 72–73 at the final follow-up, whereas the PRWE improved from 67 to 69 to 24–21. These scores surpass the pooled mean values reported by Bouri et al. [8] (MMWS: 59.9; PRWE: 31.7) and align with the findings of Charre et al. [9] and Hegazy et al. [14]. Elshahhat et al. [25] reported a DASH score of 29.4, whereas Romeih et al. [27] noted QuickDASH scores of 21.7–23.5, closely mirroring our results. Notably, no significant differences in ROM were detected between the groups, suggesting that lunate excision does not improve motion and that preservation does not impair it.

When viewed in the broader context of salvage procedures, our results demonstrate that SC arthrodesis is broadly comparable to other partial fusions. Kahve et al. [28], in patients with scaphoid nonunion who experienced advanced collapse and four-corner arthrodesis, reported that both dorsal plating and carpal screw fixation provided reliable pain relief and grip strength recovery, but screw fixation yielded superior flexion–extension arcs and earlier return to work. These findings reinforce that partial carpal fusions, whether SC or four-corner, consistently provide functional improvements, but the degree of motion preservation depends on the fixation construct and technique. Comparison with total wrist fusion underscores the value of motion-preserving procedures. Knie and van Schoonhoven [29] reported high union rates and patient satisfaction among 71 wrists at a mean follow-up of 11.7 years, with 93% of patients willing to undergo surgery again. In contrast, SC arthrodesis in our series provided durable pain relief with preservation of approximately half of the flexion–extension arc functionally sufficient for daily activities while maintaining high union rates.

From a clinical standpoint, the present findings support lunate preservation as the preferred approach during SC arthrodesis, particularly for younger or more active patients. Preservation offers more durable pain relief, maintains carpal geometry, and delays degenerative progression. Although lunate excision may provide faster early pain reduction, it is associated with greater long-term biomechanical deterioration and earlier onset of arthritic changes. Surgical decision-making should therefore be individualised, balancing short-term symptom relief against long-term joint preservation.

In conclusion, SC arthrodesis is a reliable surgical option for the management of stage IIIB Kienböck’s disease, providing significant and sustained improvements in pain, grip strength, and wrist function. When technically feasible, lunate preservation should be prioritised, as it offers superior long-term outcomes in terms of pain control, maintenance of carpal alignment, and delayed degenerative progression without compromising grip strength, the union rate, or ROM.

This study has certain limitations. Although the sample size was adequately powered to detect differences in primary outcomes, it remains relatively modest, which may limit generalisability. Retrospective registration represents a methodological limitation. Additionally, the single-centre design may have led to potential selection bias and restricted external validity. Further research in larger, multicentre cohorts—particularly those employing minimally invasive or arthroscopic techniques—is warranted to validate these findings and optimise long-term outcomes.

## Data Availability

All raw data from this study are available.
